# DeSP: a systematic DNA storage error simulation pipeline

**DOI:** 10.1186/s12859-022-04723-w

**Published:** 2022-05-17

**Authors:** Lekang Yuan, Zhen Xie, Ye Wang, Xiaowo Wang

**Affiliations:** 1 Ministry of Education Key Laboratory of Bioinformatics; Center for Synthetic and Systems Biology; Beijing National Research Center for Information Science and Technology; Department of Automation, Tsinghua University, Beijing, China 100084; 2 Tsinghua-Berkeley Shenzhen Institute, Tsinghua University, Shenzhen, 518055 China

**Keywords:** DeSP, DNA storage, Systematic error simulation, Encoding optimization, Web application

## Abstract

**Background:**

Using DNA as a storage medium is appealing due to the information density and longevity of DNA, especially in the era of data explosion. A significant challenge in the DNA data storage area is to deal with the noises introduced in the channel and control the trade-off between the redundancy of error correction codes and the information storage density. As running DNA data storage experiments in vitro is still expensive and time-consuming, a simulation model is needed to systematically optimize the redundancy to combat the channel's particular noise structure.

**Results:**

Here, we present DeSP, a systematic DNA storage error Simulation Pipeline, which simulates the errors generated from all DNA storage stages and systematically guides the optimization of encoding redundancy. It covers both the sequence lost and the within-sequence errors in the particular context of the data storage channel. With this model, we explained how errors are generated and passed through different stages to form final sequencing results, analyzed the influence of error rate and sampling depth to final error rates, and demonstrated how to systemically optimize redundancy design in silico with the simulation model. These error simulation results are consistent with the in vitro experiments.

**Conclusions:**

DeSP implemented in Python is freely available on Github (https://github.com/WangLabTHU/DeSP). It is a flexible framework for systematic error simulation in DNA storage and can be adapted to a wide range of experiment pipelines.

**Supplementary Information:**

The online version contains supplementary material available at 10.1186/s12859-022-04723-w.

## Background

In the era of data explosion, DNA emerged as a promising data storage medium due to its high information density and longevity [1]. The process of DNA storage is complicated and each stage produces noises and errors, which might lead to data corruption. Faced with this challenge, a series of error-correcting codes were introduced to recover the files from erroneous data by adding redundancy while encoding [2–6].

To find the optimal encoding method and redundancy level, the redundancy must be introduced in a proper way to combat the particular noise structure [7]. A deep understanding of the noise structure for a given DNA data storage channel is thus required for optimizing encoding methods accordingly. As running DNA data storage experiments in vitro is still expensive and time-consuming, a simulation pipeline is needed to reveal the particular noise structure of the DNA data storage channel in silico and optimize the redundancy design systematically.

To build a systematic error simulation pipeline for the DNA data storage channel, some important factors must be considered:

1. A systematic end-to-end model is desired which covers all the key stages of the storage process to reveal how errors are generated and propagated to form final sequencing results.
2. Both sequence-lost errors and errors within a sequence should be simulated.
3. When simulating within-sequence errors, the unique character of the DNA data storage process should be addressed: a sequence is stored in the pool with several copies instead of one and retrieved by combing several sequencing readouts, so the proportion of errors in the population instead of the raw error rate of a single sequencing read should be analyzed.

In the previous literature, extensive experiments were performed to characterize each stage from synthesis to Polymerase Chain Reaction (PCR) to sequencing [8–11]. These single-stage characterization studies build up the foundations for building a model for DNA data storage but can't be directly used when stages are combined. In the DNA data storage area, Hamoum put forward a channel model for the Nanopore Sequencing platform [12]. The model experts in simulating one stage accurately, but can’t capture the evolution of errors across stages in the end-to-end process systematically. For the end-to-end simulation, the sequence lost errors have been studied by Heckel and Chen [13, 14], but within-sequence errors were not well addressed. Schwarz simulated within-sequence errors in a more general context [15] but only performed simulations on a single sequence, making it not suitable for DNA data storage based on the oligo pool which consists of multiple sequences copies. By now, we still lack a systematic model that can simulate both the sequence lost and the within-sequence errors across all stages in the particular context of the DNA data storage channel.

**Fig. 1:**
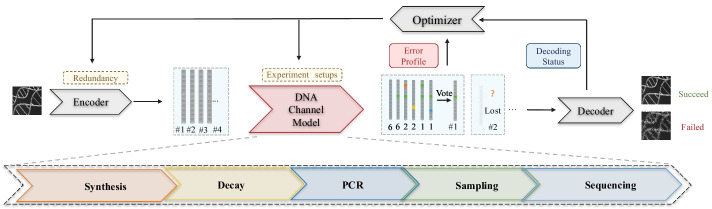
Overview of the proposed error simulation framework. A systematic error simulation model is established to model both the whole sequence lost error and the within-sequence error in the special context of DNA data storage. The model takes encoded DNA sequences as inputs and generated simulated sequencing results similar to real experiments. By analyzing the error profile and decoding status of the simulated results, redundancy of the error correction code can be optimized, providing a systematic approach for optimizing encoding designs

Here, we present DeSP, a systematic DNA storage error Simulation Pipeline. Addressing the requirements mentioned above, we established a model for the end-to-end simulation of the DNA data storage process, to reveal noise structures for a given channel quantitively. The model is flexible, as it can adapt to diverse experiment conditions, such as different sequencing platform and PCR conditions, by changing parameters accordingly and modeling different stages separately or as a whole. Based on the model's simulation functions, we put forward a method for optimizing the redundancy level systematically, finding the suitable trade-off point between information density and success probability (Fig. 1). To make the platform easy to use for the community, we provided source code, testing notebooks, and a demonstrative web application. We showed the platform can be used to generate simulated sequencing results consistent with real experiments, and demonstrated its usage for noise structure analysis and encoding optimization. With systematic in silico simulation, we hope this pipeline can boost the DNA data storage area's development, bringing a deeper understanding of the DNA data storage channel and optimal designs.

## Results

To validate the model and demonstrate its usage, we compared the simulation results against real experiment data, analyzed noise structures of DNA data storage channel using the error simulation system, and demonstrated the process of choosing proper redundancy with the encoding optimization methods.

We implemented the simulation pipeline in Python, and the package can be downloaded from https://github.com/WangLabTHU/DeSP. Users can easily construct a model fitting their needs by linking the building modules and customizing the parameters following the instructions on GitHub. The model receives DNA sequences prepared to be synthesized as input and provides raw DNA readouts as output, and we also provide functions to inspect the simulation results. A demonstrative web application is available to show how the model works (Additional file 1: Fig. S6 and Additional file 1: Note 2). Users may visit the application at the link updated on the GitHub page or deploy it locally in several minutes.

## Abstraction of the DNA data storage channel

*Data input of the simulation model:* Current technology enables the high-throughput synthesis of short oligonucleotides. To store a file into DNA library, the data must be split, indexed, and encoded into multiple DNA sequences. Therefore, the input of the DNA simulation model can be viewed as $M$ DNA sequences generated from the upstream encoding process.

*Data representation in a DNA pool:* After synthesis, one DNA sequence will have multiple copies in the DNA library pool, and errors might be introduced in those copies due to the imperfectness of the process (Additional file 1: Fig. S1). We choose such data representation accordingly: sequence $i$ has ${N}_{i}$ corresponding oligonucleotides, which is composited of $k$ error types ${e}_{i1},{e}_{i2}\dots {e}_{ik}$, with copy numbers of each error type ${n}_{i1},{n}_{i2}\dots {n}_{ik}$ as shown in Fig. 2a.

**Fig. 2:**
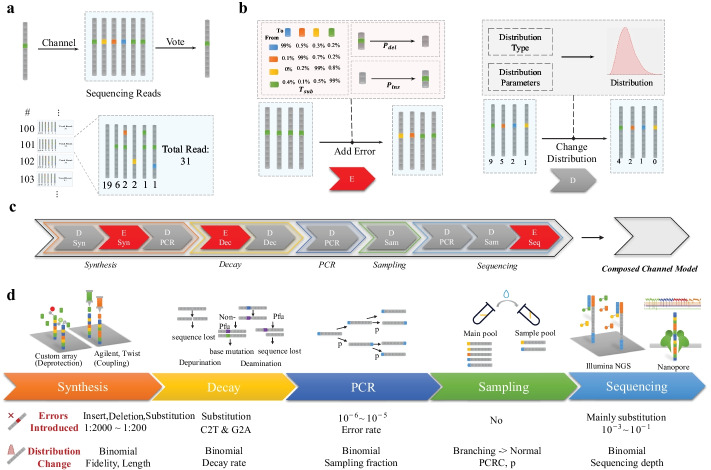
Building modules and noise source considered in DeSP. **a** Abstractions of the DNA data storage channel. A file will be split into several DNA sequences, one DNA sequence has multiple copies in the synthesized pool, and sequence information will be recovered with the voting result of multiple sequencing reads. **b** Two kinds of basic modules. E module: error generation module; D module: distribution changing module. **c** Full DNA data storage channel model built by composing basic modules. **d** A summary of how each stage influences the oligonucleotides composition in terms of introducing new errors and changing copy number distribution

*Data output and generation of voting errors:* The data is retrieved from the DNA pool by sequencing, and one sequence will have multiple sequencing readouts. The simulation model will provide the original sequencing readouts as output, following the representation similar to that in the pool (several error types with corresponding readout numbers). The raw sequencing readouts can be further combined with different methods to obtain the final consensus sequences (“Methods”). After this step, two types of errors might be produced: the whole sequence might be lost if no reads are found, and a voting error is formed if a wrong base has a stronger signal than the real base in the population.

### Combining basic modules to build a complete pipeline

To reveal the noise structures, the model needs to simulate how each stage in the data storage process influences the composition of each sequence to form the final voting errors. We abstracted two basic modules to model two kinds of influence (Fig. 2b):

*The Error Generation Module:* Errors might be introduced within a sequence when synthesis, decay, and sequencing, generating new error types: $\{{e}_{i1},{e}_{i2}\dots {e}_{ik}\}\to$ ${\{e}_{i1},{e}_{i2}\dots {e}_{ik},\dots {e}_{ik+j}\}$. We simulated the error generation process with a stochastic mutation process. The base substitution events were first simulated with possibilities controlled by substitution rate ${T}_{sub}$ (the transformation matrix, as shown in Fig. 2b. Deletion and insertion events were then simulated according to deletion rate ${P}_{del}$ and insertion rate ${P}_{ins}$. The default ${T}_{sub}$, ${P}_{del}$ and ${P}_{ins}$ for a given platform were obtained from the literature [9, 18–21].

*The Distribution changing module:* The number distribution of each error type might change in almost all stages from synthesis, decay, PCR, sampling to sequencing:${\{n}_{i1},{n}_{i2}\dots {n}_{ik}\to {n}_{i1}^{^{\prime}},{n}_{i2}^{^{\prime}}\dots {n}_{ik}^{^{\prime}}\}$.In the simulation,${n}_{ij}^{^{\prime}}$ is obtained by computing a distribution with ${n}_{ij}$ and the corresponding parameters of the given stage, and sampling a number from the distribution.

The model was built hierarchically with these two basic modules. One stage might be composed of one or more modules. Figure 2c, d provide a summary of how each stage influences the oligonucleotides composition in terms of introducing new errors and changing copy number distribution, and how this was modeled accordingly. For example, an uneven initial copy number distribution is generated when synthesizing due to the imperfect coupling efficiency, and nucleosides insertion, deletion, and substitution might occur due to the improper activation of nucleoside printing. To illustrate the two modules and the composition of modules intuitionally, we also provided an example using real simulation data in Additional file 1: Fig. S2. A more detailed description about how we modeled each stage from synthesis [9], decay [13, 16], PCR [10, 17, 18], sampling and sequencing [18–21] is provided in the Additional file 1 (Additional file 1: Fig. S1). These stages are further connected in series to form the entire DNA data storage channel (Fig. 2c).

### Comparison between simulation results and real data

To test the validity of the model, we compared the simulation data against the real data obtained from an in vitro experiment. In the experiment, we encoded a compressed file of five baseball images into 1891 DNA sequences with DNA Fountain code and synthesized them into DNA sequences by the iGeneTech oligonucleotides pool. Each DNA sequence contains 126 bases, including 64 bp (base pairs) data payload, 16 bp RS code, 16 bp seed, and 15 bp primer on both sides. The synthesized DNA sequences were then sampled, PCR amplified, and finally readout using the Illumina NextSeq500 platform.

Corresponding in silico modules were linked to build a simulation model of this process; most parameters of the simulation model were set identical to the real experiment, and some unclear parameters (such as PCR bias) were determined by parameter fitting (Additional file 1: Fig. S3). The same encoded sequences as the real experiment were passed to the established model to get simulated sequencing results under several sequencing depths, and the sequencing reads from the real experiment were also down-sampled to the same depths for comparison. According to Fig. 3, the trends of numbers of loss sequences and sequences with errors in the simulated data are consistent with the actual data. Examples of simulated sequencing readouts against actual sequencing readouts are also provided in the Additional file 1 (Additional file 1: Fig. S4).

**Fig. 3:**
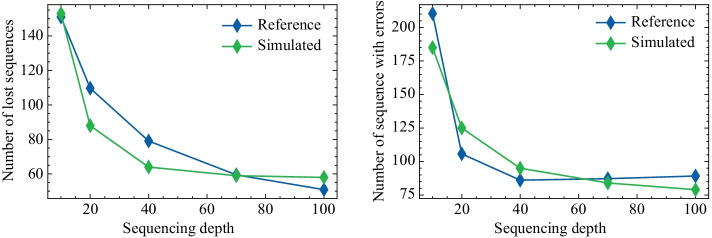
Comparison between simulation results and real data. The relationship between simulated sequencing depth and lost sequences is consistent with the experimental DNA storage sequencing result (left). The relationship between simulated sequencing depth and the number of sequences with errors is consistent with corresponding result in experimental DNA storage sequencing (right)

### Noise structures of DNA data storage channel

To reveal the DNA data storage channel’s noise structure, we used an image file of DNA double helix from the Unsplash website (“Methods”) and encode it into about two thousand strands of DNA with a length of 104 bp and passed the strands to the model. We first provided an intuitional explanation about how errors are formed by analyzing simulation results after each stage. With the observed phenomenon, we then examined how error rate, sampling, and sequencing depth influence lost number and final error rate via quantitative repeated simulations.

The distribution of oligonucleotides number in different stages are shown in Fig. 4a. The distribution started from a Binomial distribution of a synthesized pool and skewed when going through the decay and PCR process due to uneven amplification/decay ratio arising from the stochastic process. When sampling from the uneven distribution under low sampling depth, the distribution was significantly changed, and some sequences might be lost.

**Fig. 4:**
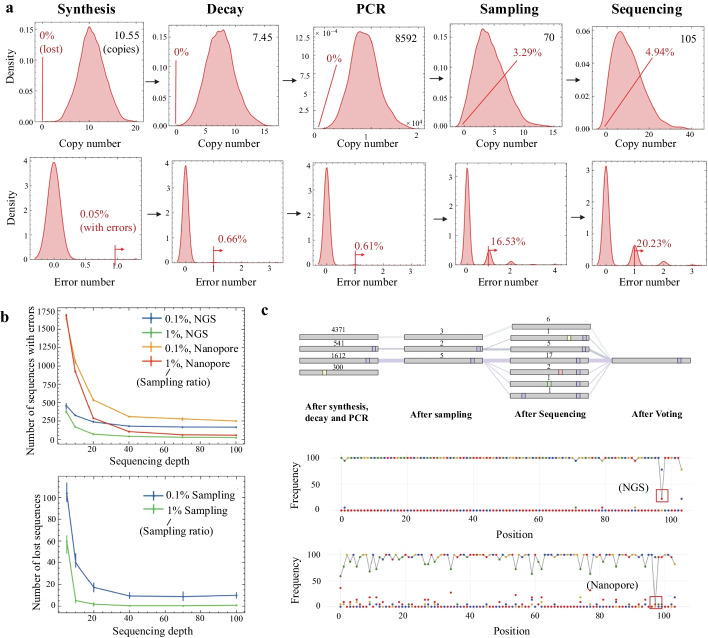
Noise structures of DNA data storage channel. **a** The Evolvement of oligonucleotides number distribution and error number distribution across the channel. The first row shows the distribution change of the copy numbers of about two thousand oligonucleotides that encode a file. A sequence will be lost if the copy number of the sequence falls to zero, and the percentages of lost sequence are denoted in the figure. In the second row, error number = 1 means the oligonucleotides sequence contains one error after voting all its copies. The percentages of sequences with more than one error are denoted. **b** Influence of sampling/sequencing depth and sequencing error rate to final voting error rate obtained from quantitative repeated experiments. **c** Copy numbers of different error types of one sequence after different stages, corresponding final voting result, and the final voting result obtained under Nanopore sequencing platform with the same sample

Sequence lost might happen both in the sampling stage and the sequencing stage. Increasing the sampling ratio would decrease the number of lost sequences (Fig. 4b). At a given sampling ratio, the number of lost sequences would decrease first with the increase of the sequencing depth, and finally coverages to a certain value. The value corresponds to the number of sequences lost in the previous stages. For example, at a 0.1% sampling rate, when the sequencing depth is higher than 40, the number of lost sequences would coverage to approximately 16, which is the number of lost sequences after sampling. According to the results, it is crucial to guarantee both the sampling ratio and the sequencing depth higher than a certain level in experiments.

### Base composition and voting error

To illustrate how final voting errors are formed, we chose one sequence and depicted the copy numbers of its different error types after passing through different stages, along with the final voting result in Fig. 4c. New errors were generated in synthesis, decay, and sequencing stages, and errors introduced in previous stages were passed to later stages when sampling. If an error base dominated in a spot, a voting error would occur.

Only changing one parameter might not lead to a change in the final error rate. For example, after replacing the sequencing method with the Nanopore sequencing, which has a much higher error rate, more errors were generated, but with enough sequencing depth, none of the new errors dominated, and there was still only one error in the final voting result.

Simulations showed that improving sequencing depth or sampling depth will lead to fewer errors, and voting errors in the sampling stage are likely to maintain in the sequencing stage. A higher sequencing error rate will lead to more errors when sequencing depth is low, but the number will decrease with increased sequencing depth to a level similar to that of the low error rate NGS platform (Fig. 4b).

### Choosing proper redundancy for encoding

For the encoding optimization part, we tested the task of choosing suitable ${L}_{RS}$ and $\alpha$ for decoding the image file of DNA double helix from the Unsplash website (“Methods”) with a successful decoding probability higher than 99%. We used Eq. (1) in the Additional file 1 to estimate information density under different ${L}_{RS}$, and ${L}_{RS}$ =2 was found to be optimal to achieve an information density of 76% (Fig. 5a).

**Fig. 5:**
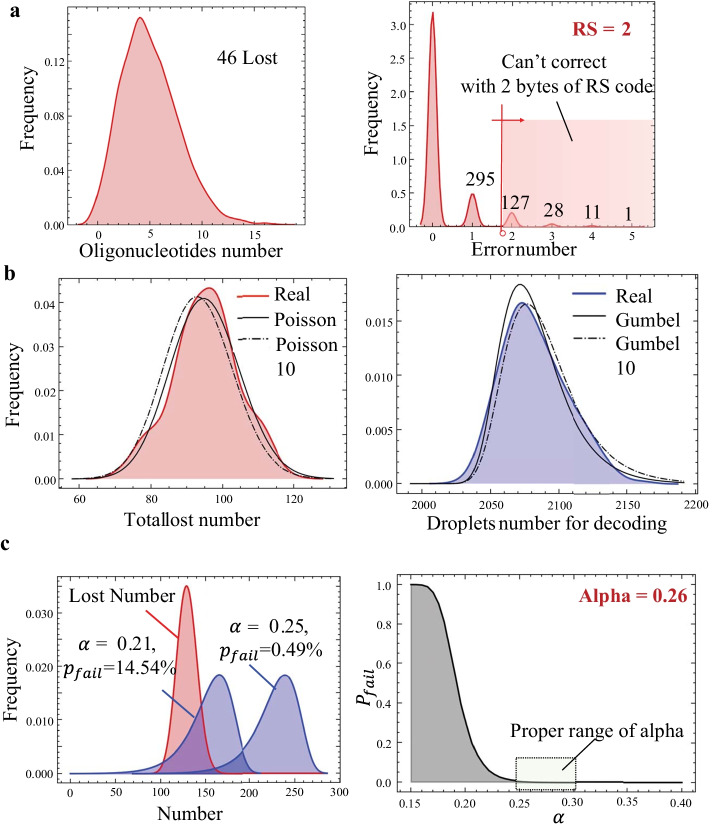
Encoding design with the model. **a** Oligonucleotides number and error number distributions of the simulated result. **b** Number distribution of total lost(left). Droplets for decoding obtained with 200 repeated experiments, and corresponding approximation computed from all the 200 experiments and 10 of the experiments(right). **c** Probability distribution of total lost (red) and permitted lost (blue) for different $\alpha$, and the relationship between failing probability and $\alpha$

Distribution types of total lost number and droplets number for successful decoding $\beta M$ are required when fitting data. With 200 repeated experiments, Poisson distribution was found to be a good approximation for total lost number distribution, possibly because every sequence has a small chance to be lost, which formed a Poisson process. The distribution for droplet numbers used for decoding is more complicated, so we empirically chose the right-skewed Gumbel distribution (Fig. 5b) that fitted the data best under the Anderson–Darling test [22].

We fitted the distributions with data obtained from 10 repeated experiments and compute the distribution of $(\alpha - \beta )M$ for different $\alpha$ with the distribution of $\beta M$, the distribution of total lost number with $\lambda = ptl(1+\alpha )N$. The failing probability was computed according to Eq. (3). Depicting the relationship between failing probability and $\alpha$ in Fig. 5c, 0.25–0.28 was found to be the acceptable range for $\alpha$.

In this experiment, the redundancy level is set optimal for a given DNA data storage channel (“Methods”). If some parts of the channel are changed, the optimal redundancy level can also be updated by rerunning the above process. For example, if we want to save some budget by choosing a lower sequencing depth, more voting errors might occur within a sequence and the total lost number ${N}_{tl}$ might increase. To guarantee the decoding probability will still be higher than 99%, we can use the above optimization methods to update the redundancy to a higher level while keeping the information density as high as possible.

## Discussion

DNA storage become an increasingly active field due to the information density and longevity of DNA. Therefore, how to control the trade-off between the information density and successful information recovery rate is essential in practice. DeSP is a systematic error simulation pipeline, which analyses how errors are generated and passed through different stages to form final sequencing results. We also demonstrated the process of choosing optimal redundancy systemically in silico before running the real experiment in vitro.

With the model, we explained how errors are generated and passed through different stages to form final sequencing results, analyzed the influence of error rate and sampling depth to final voting error rates, and demonstrated the process of choosing optimal redundancy systemically in silico before running the real experiment in vitro. We hope the model can deepen the understanding of the DNA data storage channel's noise structures and facilitate designing encoding methods for this particular noise channel.

In the future, the simulation model can be further improved in terms of efficiency and fidelity with optimized simulation algorithms and results from real experiments. With the simulation model, methods for optimizing encoding designs for other types of codes can be added under the general framework, and the optimizing targets can be extended from the encoding system to both the encoding system and the data channel, optimizing encoding success probability, information density and experiment cost.

## Conclusions

DeSP is a systematic pipeline that can simulate both the sequence lost and the within-sequence errors across all stages in the particular context of DNA data storage. Most importantly, the simulation pipeline helps to deepen the understanding of the DNA data storage channel's noise structures and facilitate designing encoding methods for this particular noise channel. DeSP also provides an easy-to-use demonstrative web application version for diverse users.

## Methods

### Flexibility and expansibility of the model

*Modularity brings flexibility for using the model.* Using the simulation model, users may pass the data through separate stages to explore what happens in different stages to understand the DNA data storage channel (Fig. 3a, Additional file 1: Fig. S5). They can also use a composed model to examine how different parameters influence the final error rate.

*The model also enjoys high expansibility.* It can be configured to model various experiment setups, serves as a general simulation framework for the DNA data storage area. Users can choose individual modules and link them properly (Fig. 2c), assign parameters to selected modules, and even create a new module without changing other modules. With a minor modification of the code, users can establish a whole model to suit their needs.

The established model can also be embedded in various encoding–decoding pipelines. The in silico simulation model shares the same data input and output with the real experiment process: it receives encoded DNA sequences as input and generates raw erroneous copies for each sequence as outputs. As no assumptions are made about the external encoding/decoding modules when building the simulation model, it can receive encoded sequences from any encoding methods as input. The raw output can be combined with different clustering/consensus methods to obtain retrieved sequences, which are then passed to the decoding module to retrieve the original data. Decoupling with external modules brings more flexibility to the model, enabling it to serve as a general simulation platform in a wide range of experiment pipelines.

### Encoding design optimization principles in DNA storage channel

Noise might be generated in the storage process, causing data corruption. Introducing redundancy with certain encoding methods could combat the noise with the cost of low efficiency. An ideal encoding design should be suitable for the storage process’ noise structure, with minimum redundancy just enough to combat the noise level. This part will discuss how to choose the optimal redundancy level for a given channel with the model.

The optimal redundancy should be set to a proper level to balance two goals: a. maximizing information density (the number of bits stored in one base). b. maximizing the probability for successful data retrieval. Two factors are needed to compute the success probability: noise level of the channel (how many errors might be generated in a given channel) and error-correction ability of the code (how many errors can be corrected with the introduced redundancy). The model can be used to estimate both factors to compute the relationship between information density and probability. Then the redundancy level can be easily selected to meet a specific need.

**Fig. 6:**
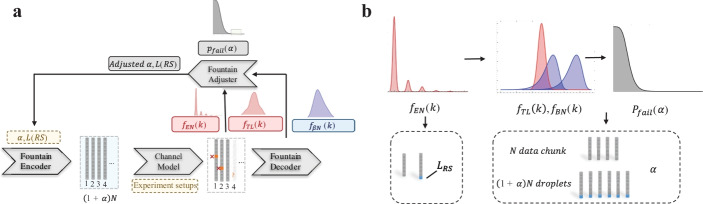
Optimizing redundancy for the encoding method including Fountain code and RS code. **a** The error rate was simulated by DeSP and guided the adjustment of parameter α and ${L}_{RS}$. **b** The target optimization parameter α determines how many extra sequences are generated when encoding with the fountain code. The parameter ${L}_{RS}$ is the length of the RS code

Here, we provide a case for choosing the proper redundancy level for the DNA fountain code [5] as an example (Fig. 6). We used an image file of DNA double helix from the Unsplash website (https://unsplash.com/license) and resized it into 9 KB. Methods can also be developed for other types of code to find optimal redundancy levels with the general simulation model.

### Choose redundancy level for DNA Fountain code

DNA Fountain code is one of the most popular encoding methods used in the DNA data storage area. It uses fountain code to deal with the sequence lost error and RS (Reed-Solomon) code to combat the within-sequence errors. The redundancy level of DNA Fountain code is related to the parameter of the fountain code, $\alpha$, and the length of the RS code ${L}_{RS}$.

The parameter $\alpha$ determines how many extra sequences are generated when encoding with the fountain code. When encoding, data are divided into $M$ chunks and encoded into $(1+\alpha )M$ droplets, which are then synthesized into DNA. To recover the data, only $(1+\beta )M$ droplets are needed, which permits $(\alpha -\beta )M$ sequences to be lost. Increasing $\alpha$ will provide higher tolerance to the sequence loss, but will also lead to a lower information density.

The parameter ${L}_{RS}$ means how many bytes of RS code are added within the sequence. Before decoding the fountain code, we need to make sure every droplet used for decoding is errorless, so we need to correct the within-sequence errors of each droplet. This is achieved by adding RS code to the generated droplets while encoding to protect a sequence from within-sequence noise under a certain level: by adding ${L}_{RS}$ bytes of RS code, $\left[{L}_{RS}/2\right]$ bytes of error can be detected and corrected. When decoding, for each sequence, if the sequence contains no error or the error can be corrected with the RS code, it will be passed to the fountain code. Otherwise, it will be discarded, leading to the same effect as sequence lost, and the total lost number ${N}_{tl}$ can be calculated by adding the numbers of lost and discarded sequences together. Similar to $\alpha$, longer ${L}_{RS}$ means higher tolerance to within-sequence errors but lower information density. Note there is a correlation between $\alpha$ and ${L}_{RS}$: if ${L}_{RS}$ is set higher, sequences with more errors can also be corrected and we will have more valid droplets for decoding, so smaller $\alpha$ might be enough to deal with the fewer total droplets lost.

### Optimizing $\boldsymbol{\alpha }$ and ${{\varvec{L}}}_{{\varvec{R}}{\varvec{S}}}$ with the model

When trying to obtain the highest information density while guaranteeing a certain level of success probability, both ${L}_{RS}$ and $\alpha$ should be optimized.

${L}_{RS}$ can be optimized first according to error number distribution. As an intuitive example, among the sequences with errors, if most sequences contain only one error, it might be a wise choice to set ${L}_{RS}$ = 2 to correct only one error, as discarding sequences with more than one error won’t bring too much burden on $\alpha$.To choose ${L}_{RS}$ quantitatively, information densities $D(k)$ under different RS code lengths $k$ can be estimated. The overall information density is a combination of the within-sequence information density (the left part, in which ${L}_{d}$ denotes the length of the data in bytes, $k$ denotes the length of the RS code in bytes) and the across-sequence information density (the right part, in which $N$ is the number of data chunks before encoding, $(1+\beta )N$ is the number of droplets needed for decoding, ${N}_{l}$ is the number of the lost sequences, and ${\sum }_{i=k}^{\infty }{N}_{e}(i)$ is the number of sequences that contain too many errors for the RS code to correct and are abandoned.

From Eq. (1), we can also see the trade-off when choosing the proper ${L}_{RS}$: if we want to obtain a high within-sequence information density by setting RS code length $k$ low, the number of sequences that can not be corrected ($\sum_{i=k}^{\infty }{N}_{e}\left(i\right)$) will increase, so more droplets need to be generated, leading to a low across-sequence information density. According to Eqs. (1) and (2), ${L}_{RS}$ was chosen to obtain the highest information density. The parameter $\alpha$ was then optimized under a given ${L}_{RS}$.

To choose proper $\alpha$, the relationship between the total lost number ${N}_{tl}$ and permitted lost number $(\alpha -\beta )M$ should be considered: only when $(\alpha -\beta )M$ >${N}_{tl}$ the data can be recovered. ${N}_{tl}$ and $(\alpha -\beta )M$ are not constants in different trials, both follow certain distributions, which can be computed by estimating parameters of a certain type of distribution with samples from 5–10 repeat experiments.

In detail, the distribution functions are obtained by the following two steps:

1. Obtaining the prior about the distribution family. The distribution prior can be determined with theoretical derivation or empirically with experimental results. Here, we found ${f}_{{N}_{tl}}(k)$, follows the Poisson distribution, and ${f}_{(\alpha -\beta )M}(k)$, follows the right-skewed Gumbel distribution by repeated experimental results, as discussed in the second paragraph in part “Choosing proper redundancy for encoding” and Fig. 5b.
2. Fitting the parameters of the distribution with data obtained from 10 repeated experiments. For example, for ${f}_{{N}_{tl}}(k)$, sequence lost numbers of 10 repeated simulations are calculated to fit the λ of the Poisson distribution.

With both distributions ${f}_{{N}_{tl}}(k)$ and ${f}_{\left(\alpha -\beta \right)M}(k)$,the relationship between the failing probability ${p}_{f}$ and $\alpha$ can now be calculated following Eq. (3). The decoding will fail if the lost number $i$ is higher than the number of droplets permitted to be lost $j$:

1 $$D(k) = \left( {\frac{{L_{d} }}{{L_{d} + 2k}}} \right)\left( {\frac{N}{{(1 + \beta )N + N_{l} + \sum\nolimits_{i = k}^{\infty } {N_{e} (i)} }}} \right)$$

2 $$L_{RS} = 2\,argmax[D(k)]$$

3 $$p_{f} (\alpha ) = \sum\limits_{j = 0}^{{T_{max} }} {\sum\limits_{i = 0}^{j} {f_{{N_{tl} }} (i)f_{(\alpha - \beta )M} (j)} }$$

The value of $\alpha$ can be determined accordingly to make the tradeoff between information density and success probability optimal for the application.

## Supplementary Information

**Additional file 1.** Supplementary methods, figures and user guideline for DeSP.

## Data Availability

DeSP implemented in Python is freely available on Github (https://github.com/WangLabTHU/DeSP).
