# Correction to: DeSP: a systematic DNA storage error simulation pipeline

**DOI:** 10.1186/s12859-022-04813-9

**Published:** 2022-07-12

**Authors:** Lekang Yuan, Zhen Xie, Ye Wang, Xiaowo Wang

**Affiliations:** 1 Ministry of Education Key Laboratory of Bioinformatics; Center for Synthetic and Systems Biology; Beijing National Research Center for Information Science and Technology; Department of Automation, Tsinghua University, Beijing, 100084 China; 2 Tsinghua-Berkeley Shenzhen Institute, Tsinghua University, Shenzhen, 518055 China

## Correction to: BMC Bioinformatics (2022) 23:185 https://doi.org/10.1186/s12859-022-04723-w

Following the publication of the original article [1], the authors identified that Ye Wang was not assigned as the co-corresponding author, and Lekang Yuan was not affiliated with affiliation 1.

The author group has been updated above and the original article [1] has been corrected.
