# Supplementary material for: DeSP: a systematic DNA storage error simulation pipeline
Source: BMC Bioinformatics. 2022 May 17;23:185. doi: 10.1186/s12859-022-04723-w (PMC9116035; doi:10.1186/s12859-022-04723-w)
Supplement: Supplementary file 1 — Additional file 1. Supplementary methods, figures and user guideline for DeSP. [file 12859_2022_4723_MOESM1_ESM.docx]

**Suppl. Fig. 1**

**
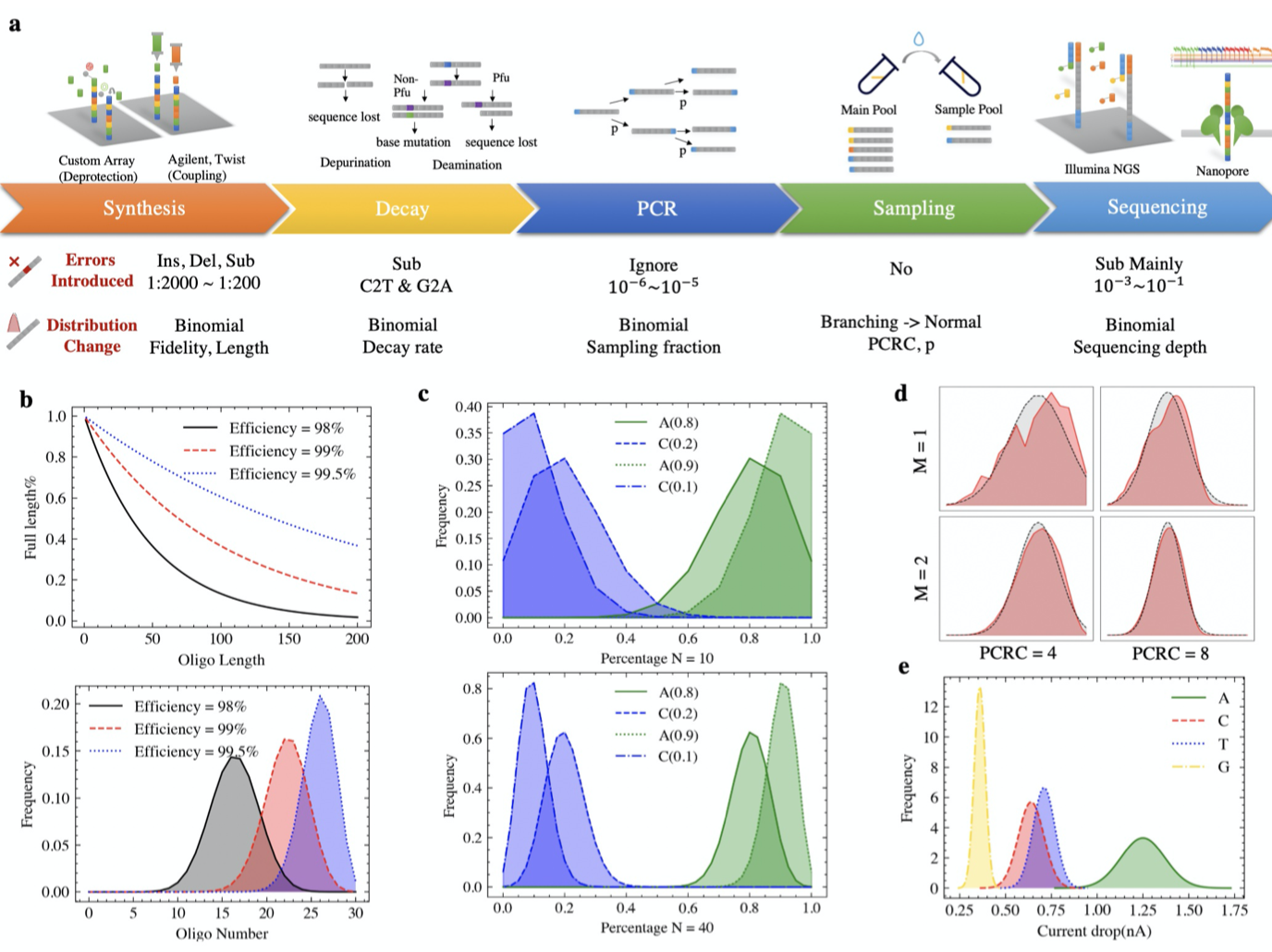
**

Suppl. Fig. 1: Principles and error sources of individual stages. a. Principle diagrams and summary of error sources of different stages in DNA data storage channel. b, Relationship between coupling efﬁciency, oligo length with the percentage of successfully synthesized oligos, and number distribution of synthesized oligos. c, Percentage distribution after sampling. 10 or 40 copies are sampled from two pools of different error rates (80% A and 20% C, 90% A and 10% C, A is the actual base), and the probability distribution of percentages of two bases is depicted. Substitutions may occur in the overlap areas. d, Distribution after PCR with different initial numbers $M_{0}$ and PCR cycles under p = 0.8. f, Current drop distribution of four bases in Nanopore sequencing. Substitutions may occur in overlap areas.

**Suppl. Fig. 2**

**
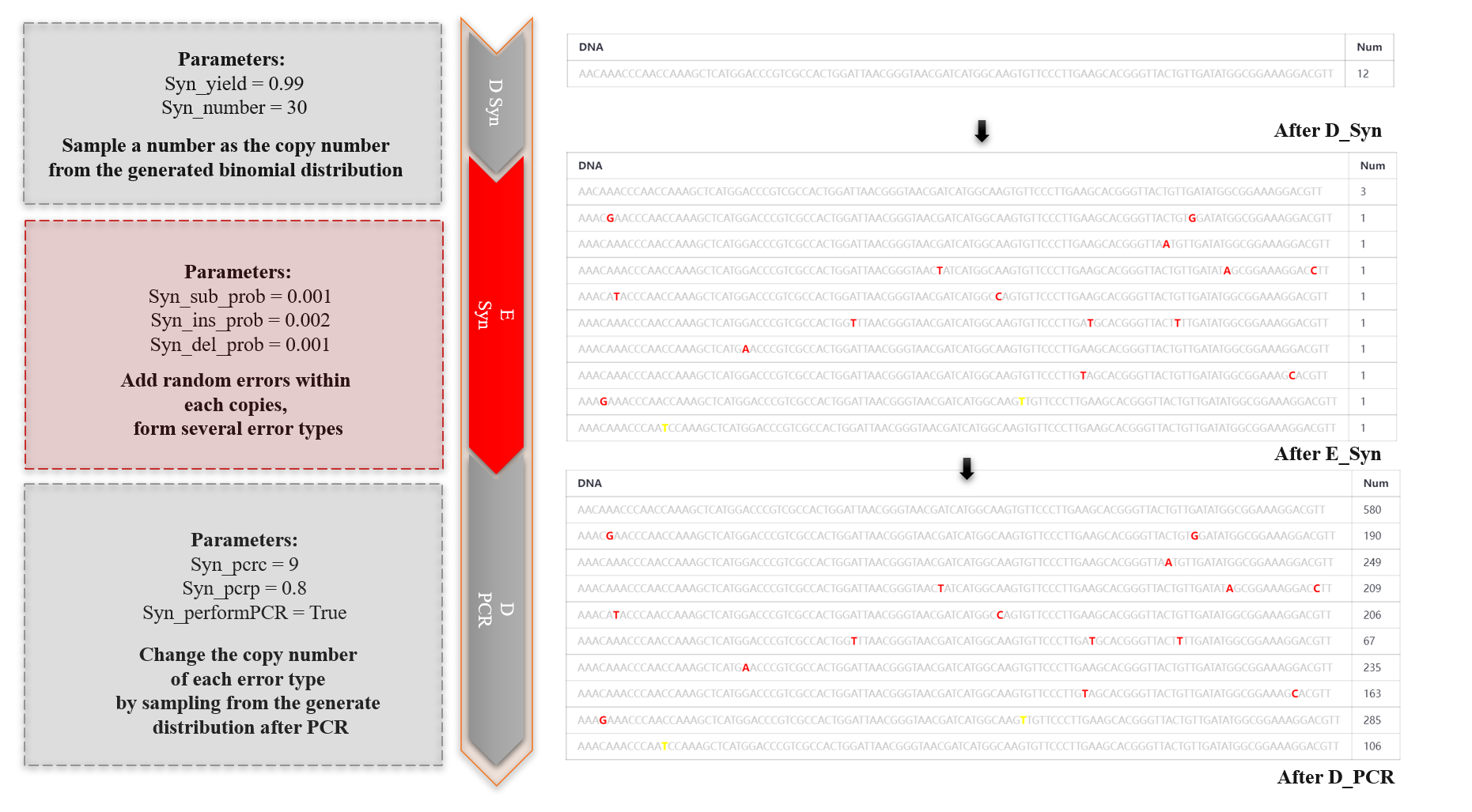
**

Suppl. Fig. 2: An example of how the synthesis stage model works. The error module adds new within-in sequence errors, and the distribution module changes the copy number distribution of existing error types. One stage might be composed of several basic error modules and distribution change modules. The base compositions and copy numbers of all error types of a sequence after each block are shown on the right.

**Suppl. Fig. 3**


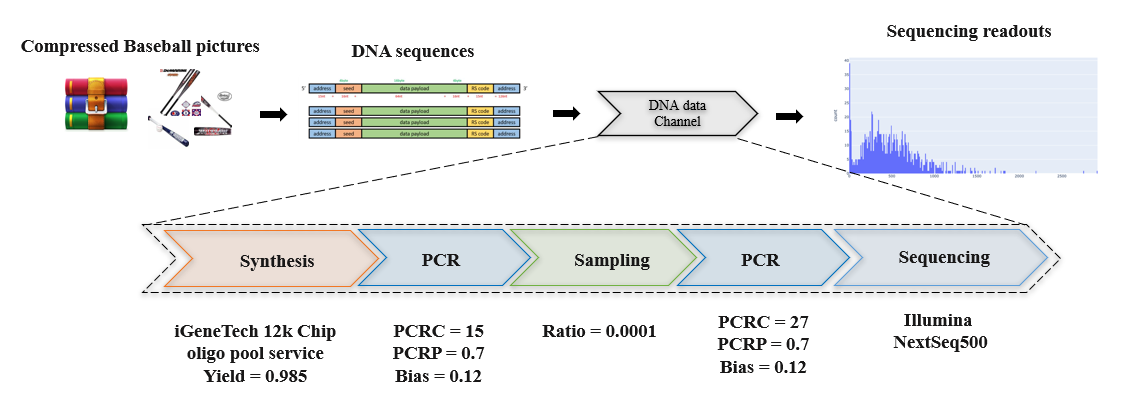


Suppl. Fig. 3: *In vitro* experiment setup. A file encoded into 1891 DNA sequences was chosen to perform the comparison analysis.

**Suppl. Fig. 4**

**
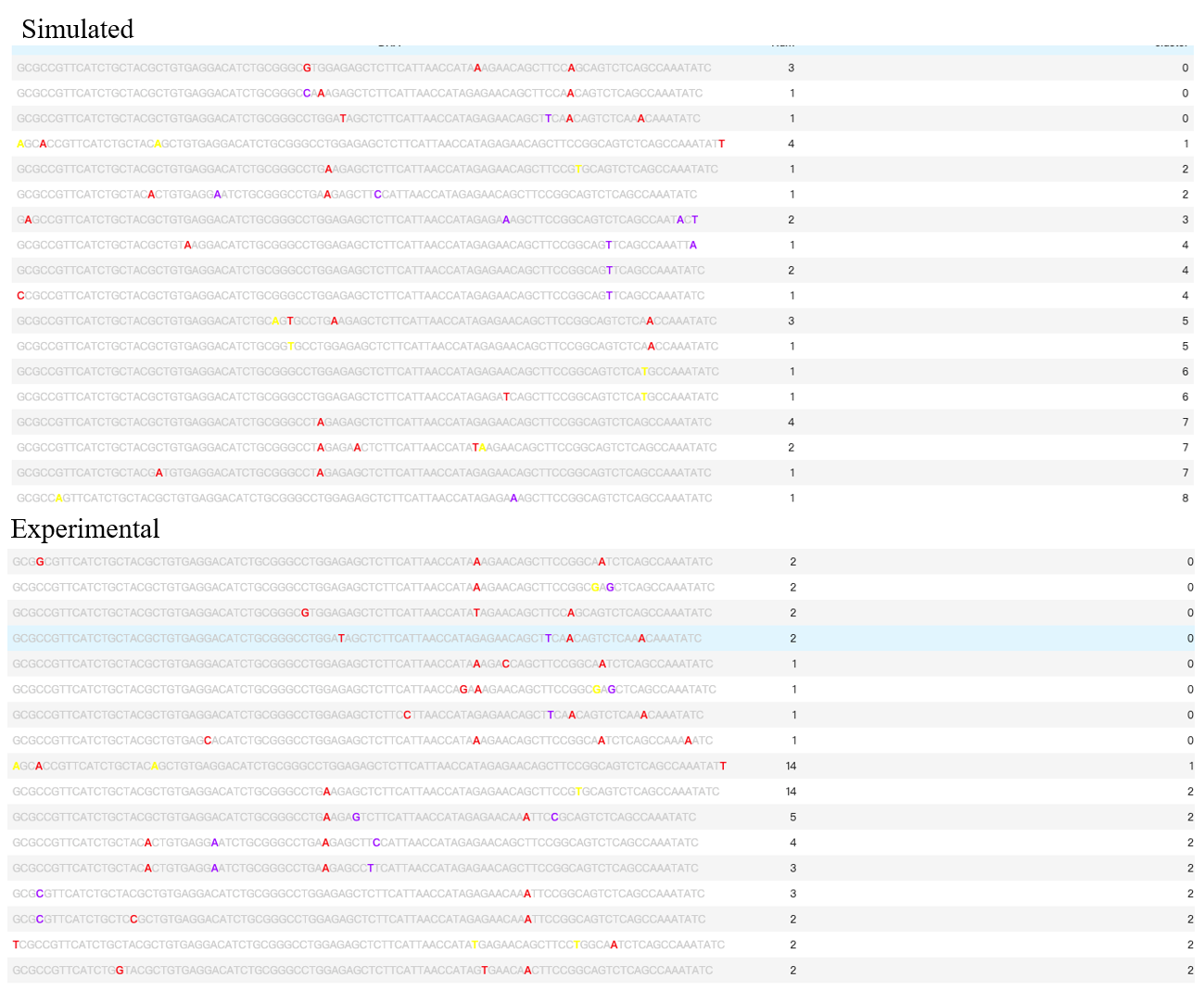
**

Suppl. Fig. 4: Simulated and actual sequencing readouts of one DNA sequence.

**Suppl. Fig. 5**

**
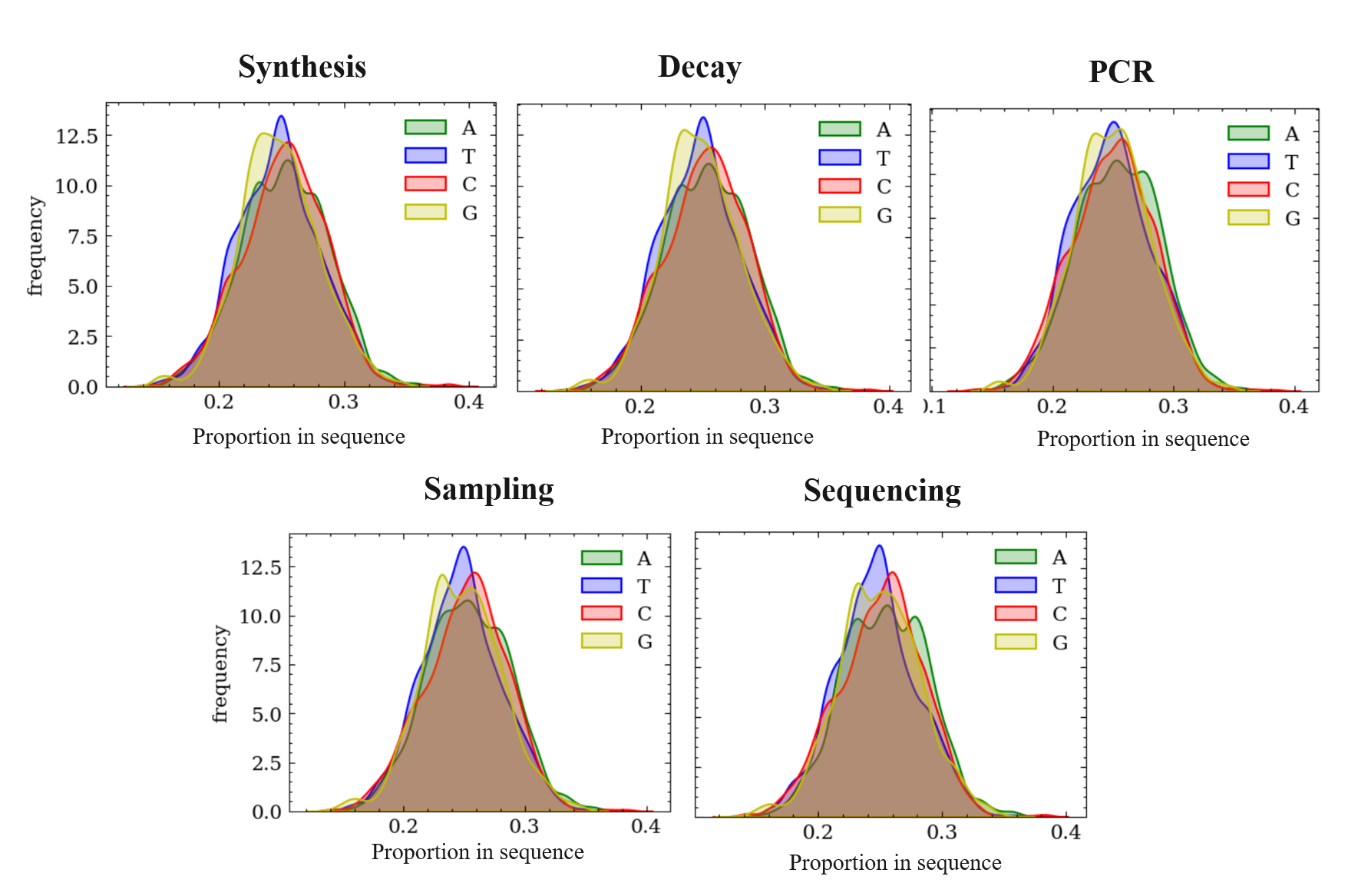
**

Suppl. Fig. 5: The distribution change of four nucleotides across the simulation. This figure shows the distribution of the proportion of each nucleotide in all sequences. The nucleotides distribution is mostly determined after encoding, and will only fluctuate slightly in the subsequent stages.

**Suppl. Fig. 6**

**
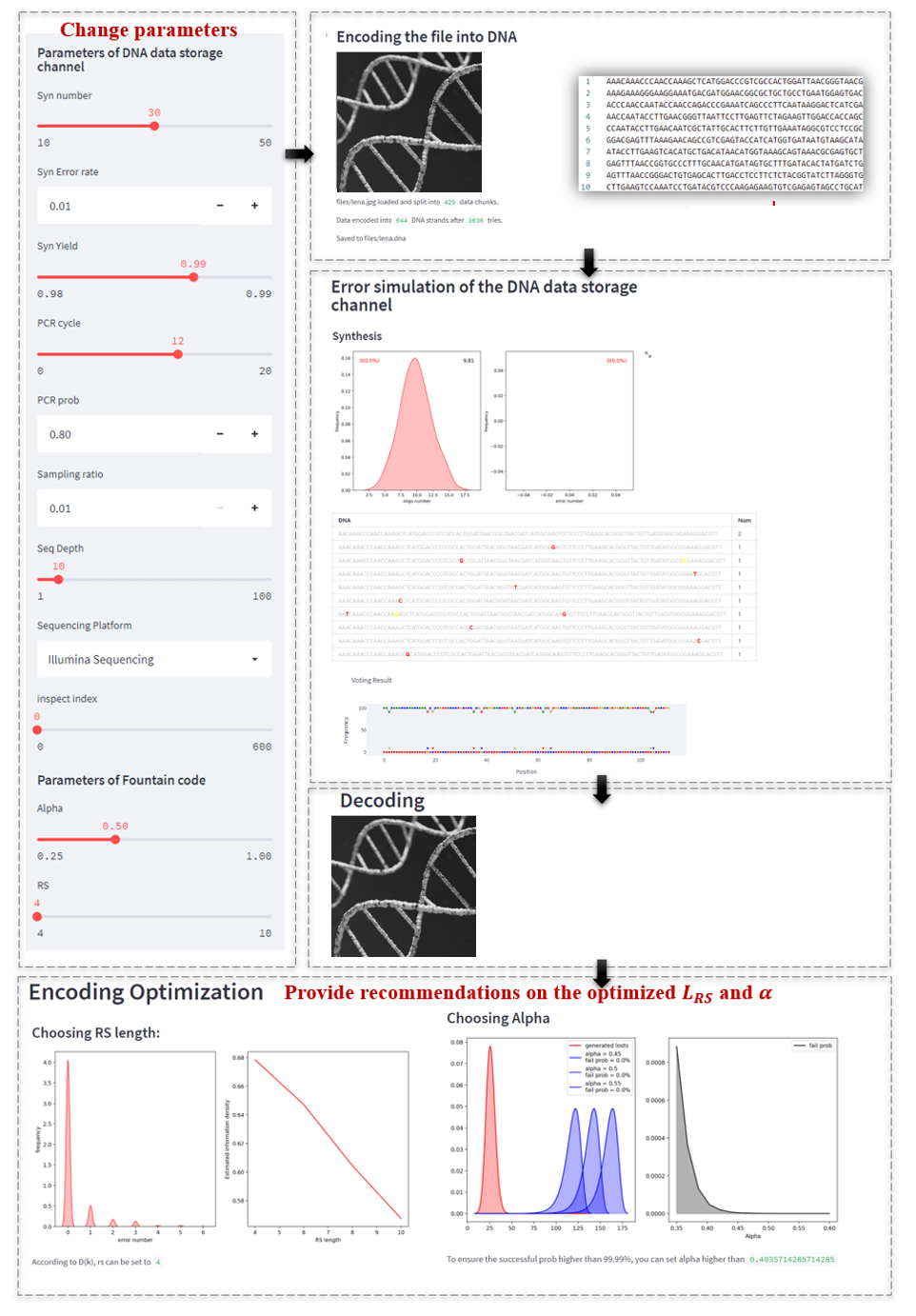
**

Suppl. Fig. 6: The web application. Steps to use the web application are explained in the Suppl. Note. 2.

**Suppli. Note 1 Analysis of individual stages**

1. **Synthesis**

*Principles of DNA synthesis:* Synthesis is the ﬁrst step in DNA data storage. Most synthesis techniques are based on phosphoramidite chemistry, where bases are added to the current sequence serially in a three stages synthesis loop [1-2]. To get high throughput, oligos (short DNA strands) are synthesized in parallel in different spatial locations on a chip, so to add the speciﬁc base to a speciﬁc location, at least one step of the synthesis loop must be controlled spatially. This can be done by controlling the deprotection step with light or electric activation or controlling the coupling step by printing desired nucleosides to appropriate locations (Suppl. Fig.1b). After the extension process is done, synthesized oligos are cleaved, deprotected, and PCR ampliﬁed to form an oligo pool.

*Synthesis errors and number distribution:* In the synthesis process, nucleosides insertion, deletion, and substitution might occur due to improper activation or nucleoside printing, with error rates ranging from 1:2000 to 1:200. Moreover, the synthesis process might also be terminated at each step because of unsuccessful coupling, with the possibility of adding one nucleoside to the current strand successfully deﬁned as coupling efﬁciency, typically about 98-99.5. As shown in Suppl.Fig.1b, the imperfect coupling efﬁciency limits the length of the synthesized sequence as the possibility of getting a complete sequence will decrease exponentially with the increase of sequence length. The number distribution of successfully synthesized oligos will also become uneven due to this stochastic process, and the generated distribution can be theoretically modeled as a binomial distribution.

*Modeling methods:* We modeled the synthesis process as synthesizing $M$ oligos of length $L$. The synthesized number of each oligo $n_{i}$ was obtained by sampling from a distribution computed with coupling efﬁciency $P_{c}$ and length $L$, and within-sequence errors were then added following the error rate of a speciﬁc platform.

1. **Decay**

*Principle and inﬂuence:* Synthesized DNA strands decay in the storage process due to two reactions: depurination and deamination. Depurination generates AP sites (apurinic/apyrimidinic site) that undergo spontaneous cleavage, leading to strand break and thus sequence loss; deamination causes C2U mutation, leading to G2T mutation when using proofreading enzymes, or sequence loss using proofreading enzymes [1].

*Modeling methods:* The depurination and deamination ratios were computed with temperature, PH, and storage time following equations described in the literature [3]. Users can also directly deﬁne the ratios. If proofreading enzymes are used, the deamination ratio will be added to the sequence loss ratio. Otherwise, G2T errors will be added to the sequence. The number $n_{ij}$ of error type $e_{ij}$ was altered following a binomial distribution, as each oligo has a possibility $p_{loss}$ to be lost.

1. **PCR Ampliﬁcation**

*Distribution changed in the branching process:* PCR ampliﬁcation is widely used throughout the process after synthesis, sampling, and sequencing. In each PCR cycle, a sequence has a possibility of $p$ to be ampliﬁed, deﬁned as replication efﬁciency. In the context of DNA data storage, we ignored the saturation effect and sequence dependency effect [4-6] as the reaction will mainly remain in the exponential stage, and primer sequences are the same for all oligos encoded from the same ﬁle and treated replication efﬁciency as a constant throughout the reaction process for every sequence.

*Modeling methods:* The process can thus be modeled as a branching process. Given initial number $N_{0}$ and ampliﬁcation possibility $p$, the number of a sequence of the next cycle $N_{n+1}$ has a relationship with that of the previous cycle described in (1), with mean $u_{N}$ and variance $\sigma_{N}^{2}$ following (2) and (3):

$$\begin{aligned} N_{n+1}=N_{n}+B\left( N;p \right) \#(1) \end{aligned}$$

$$\begin{aligned} u_{N}=M_{0}\left( 1+p \right)^{n} \#(2) \end{aligned}$$

$$\begin{aligned} \sigma_{N}^{2}=N_{0}\frac{1-p}{1+p}\left[ \left( 1+p \right)^{2n}-\left( 1+p \right)^{n} \right]\#(3) \end{aligned}$$

Copy number of error type $e_{ij}$ after PCR was sampled from a distribution computed with the initial number $n_{ij}$, replication efﬁciency $p$, and PCR cycles $n$. As the actual distribution is costly to compute, we used Normal distribution as an approximation, which introduced neglectable bias under typical PCR cycles, as shown in Suppl.Fig.1d. As PCR is a high ﬁdelity process (with typical error rates in the order of ${10}^{-6}$ to ${10}^{-5}$ ) [7], within-sequence errors introduced in this stage were ignored in our model.

1. **Sampling**

*Sampling leads to sequence loss and change in the error sequence ratio:* A small fraction of DNA must be drawn from the main oligo pool to read or copy the data, and when sequencing, only random oligos are sequenced. These can be modeled as a random sampling process. The sampling process has a substantial impact on the error characteristic: a sequence will be lost if no copies of it are sampled; sampling may change the base composition of a speciﬁc spot, and a voting error will occur if a wrong base has more copies than others in the population.

The inﬂuence of original error rates and sampling depth on the possibility of a voting error can be illustrated in Suppl.Fig.1c. Imaging a fraction of copies of a sequence in the original pool contains a base mutation in a spot. Suppose $p_{err}$ is the ratio of error sequences in the pool. The numbers of the right base and the wrong base follow certain distribution when sampling $N$ samples of that sequence. The error rate $p_{err}$ determines the mean of the distribution, and sampling depth $N$ inﬂuences the distribution variance. Both higher mean and higher variance of the percentage distribution of a wrong base will lead to a higher possibility that a wrong base will be observed more frequently than the real base.

*Modeling methods:* When drawing a certain percentage of drops from the pool, each oligo has a possibility $p_{s}$ to be sampled, leading to a Binomial distribution of each error type. $p_{s}$ equals the sampling percentage, which should be deﬁned by the user.

1. **Sequencing**

*Principles of DNA sequencing:* To read the data out, sequencing is needed. NGS (Next-generation sequencing) sequencing platform is the most commonly used technique for now, as it is inexpensive, accurate, and has high data throughput. In the sequencing process, oligos are immobilized to a solid surface and ampliﬁed to form clusters to increase signal strength. Then, dye-labeled modiﬁed nucleotides are added to each cluster one base at a time according to the template so that the template base can be determined via imaging [8]. Nanopore Sequencing [9] is also an appealing sequencing method developed in recent years. In this method, a base is determined by the current drop it generates when passing through a nanoscale pole.

*Sequencing errors:* Substitution is the primary error type when sequencing. This happens because of insufﬁcient discrimination of the emitted signal of the respective base, either light emitted by different attached ﬂuorophore or current drop, later shown in Suppl.Fig.1e. Certain substitutions are more likely to happen than others due to large overlap in signal probability distributions, such as A2C in Illuminate NGS platform or A2G in Nanopore sequencing [9-11].

*Modeling methods:* We modeled this stage as drawing $MD$ strands from the pool and adding errors to these strands to form the ﬁnal sequencing result. $M$ is the number of sequences, and $D$ is the sequencing depth. Error rates for a speciﬁc platform are obtained from literature and company websites [8-12]. Users can also use customized error rates.

**Suppl. Note. 2 Steps to using the web application.**

*Step1: Modeling a specific pipeline by changing the channel parameters.*

Users can change the channel parameters on the left panel to model a specific experimental setup. They can also use the default parameters to try a typical setup.

Besides channel parameters, there are also some other parameters on the left panel:

1. Inspect index: which sequence to inspect in detail during the simulation process.
2. $\alpha$ and $RS$: initial redundancies for the error correction code used when encoding.

*Step2: Encode, error simulation, and decode.*

After modifying the parameters, the web application will run the whole DNA data storage pipeline from encoding to decoding.

*Encode:* The Web application will first encode the input file into a .dna file, which contains all the sequences to be synthesized. Redundancy is added according to the $\alpha$ and $RS$ on the left panel.

*Error simulation:* The simulation pipeline takes the .dna file as input. For each stage, three figures will be depicted: Oligo copy number distribution and voting error number distribution; Error types of sequence chosen with Inspect Index; Voting results of current copies of sequence chosen with Inspect index. Users can check those figures to see how error evolves across stages. The output of the simulation pipeline is the voting result of the sequencing readouts, which are saved in a .dna file.

*Decode:* The decoding process takes the simulated .dna file as input and tries to decode the data.

*Step3: Redundancy optimization*

Finally, with the methods described in section 2.4.3, the web application will predict the relationship between the redundancy levels and the successful retrieval possibility and recommend an optimized redundancy level to obtain the information density as high as possible while ensuring a desired successful possibility.

**References**

1. Heckel, R., Mikutis, G., and Grass, R. N. (2019). A characterization of the dna data storage channel. Scientiﬁc reports, 9(1), 1–12.
2. Kosuri, S. and Church, G. M. (2014). Large-scale de novo dna synthesis: technologies and applications. Nature methods, 11(5), 499-507.
3. An, R., Jia, Y., Wan, B., Zhang, Y., Dong, P., Li, J., and Liang, X. (2014). Nonenzymatic depurination of nucleic acids: factors and mechanisms. PloS one, 9(12), e115950.
4. Pan, W., Byrne-Steele, M., Wang, C., Lu, S., Clemmons, S., Zahorchak, R. J., and Han, J. (2014). Dna polymerase preference determines pcr priming efﬁciency. BMC biotechnology, 14(1), 1–17.
5. Jagers, P. and Klebaner, F. (2003). Random variation and concentration effects in pcr. Journal of Theoretical Biology, 224(3), 299–304.
6. Dohm, J. C., Lottaz, C., Borodina, T., and Himmelbauer, H. (2008). Substantial biases in ultra-short read data sets from high-throughput dna sequencing. Nucleic acids research, 36(16), e105.
7. Cline, J., Braman, J. C., and Hogrefe, H. H. (1996). Pcr ﬁdelity of pfu dna polymerase and other thermostable dna polymerases. Nucleic acids research, 24(18), 35463551.
8. Metzker, M. L. (2010). Sequencing technologies—the next generation. Nature reviews genetics, 11(1), 31–46.
9. Yang, H., Li, Z., Chen, Y., and Si, W. (2017). Identiﬁcation of single nucleotides in sin nanopore. Biophysical Journal, 112(3), 25a.
10. Ross, M. G., Russ, C., Costello, M., Hollinger, A., Lennon, N. J., Hegarty, R., Nusbaum, C., and Jaffe, D. B. (2013). Characterizing and measuring bias in sequence data. Genome biology, 14(5), 1–20.
11. Schirmer, M., D’Amore, R., Ijaz, U. Z., Hall, N., and Quince, C. (2016). Illumina error proﬁles: resolving ﬁne-scale variation in metagenomic sequencing data. BMC bioinformatics, 17(1), 1–15.
12. Weirather, J. L., de Cesare, M., Wang, Y., Piazza, P., Sebastiano, V., Wang, X.-J., Buck, D., and Au, K. F. (2017). Comprehensive comparison of paciﬁc biosciences and oxford nanopore technologies and their applications to transcriptome analysis. F1000Research, 6-100.
